# Editorial: Vascular Inflammation in Systemic Autoimmunity

**DOI:** 10.3389/fimmu.2016.00471

**Published:** 2016-11-21

**Authors:** Giuseppe A. Ramirez, Cornelia Weyand, Augusto Vaglio, Angelo A. Manfredi

**Affiliations:** ^1^Unit of Internal Medicine and Immunology, IRCCS Ospedale San Raffaele, Milan, Italy; ^2^Università Vita-Salute San Raffaele, Milan, Italy; ^3^Department of Medicine, Division of Immunology and Rheumatology, Stanford University, Stanford, CA, USA; ^4^Unit of Nephrology, University Hospital of Parma, Parma, Italy

**Keywords:** vascular inflammation, autoimmunity, vasculitis, systemic inflammation, remodeling

**The Editorial on the Research Topic**

**Vascular Inflammation in Systemic Autoimmunity**

The immune system evolutionarily arises to integrate distant tissues and coordinate responses toward environmental or endogenous threats in complex living beings (1). To this purpose, innate and adaptive immune cells exploit the vast network provided by the vasculature, which enables information exchange and physical communication between threatened tissues and tissues in charge of immune cell generation and maturation as well as metabolism regulation (2). Clinical evidence of vascular inflammation can be easily found in settings of persisting inflammation and autoimmunity. Conversely, inflammation is a hallmark of disease progression in metabolic and cardiovascular diseases, which are the major causes of morbidity and mortality in the general population (3). If we translate these evidences into a prospect toward the future of research and clinical practice in immunology, two main questions arise:

- Can we identify shared pathogenic events among apparently unrelated human diseases that lead to vascular inflammation?
- Can we tackle these events to improve patient diagnosis, risk stratification, and treatment?

Although we are still far from satisfactorily answering these questions, here we highlight some of the emerging connections between vascular inflammation and systemic autoimmunity.

The first set of articles draws attention to some emerging players in the development and the maintenance of vascular inflammation (Figure 1). Khaib Dit Naib et al. report on the role of IL10 promoter variants in the susceptibility to Behçet’s disease, adding evidence to the postulate of a common pathogenic background between Behçet’s disease and other autoinflammatory conditions, such as Crohn’s disease (4–6), where platelet activation and smoldering vascular inflammation may occur (7, 8). Additional hints on common genetic patterns of susceptibility among diseases characterized by vessel inflammation are emerging from multicenter studies (9). Bonatti et al. and Pattanaik et al. provide a thorough and updated appraisal on this topic by analyzing two paradigmatic conditions: anti-neutrophil cytoplasmic antibodies-associated vasculitides and systemic sclerosis, the former being characterized by necrotizing vessel inflammation, the latter by chronic vessel remodeling.

**Figure 1:**
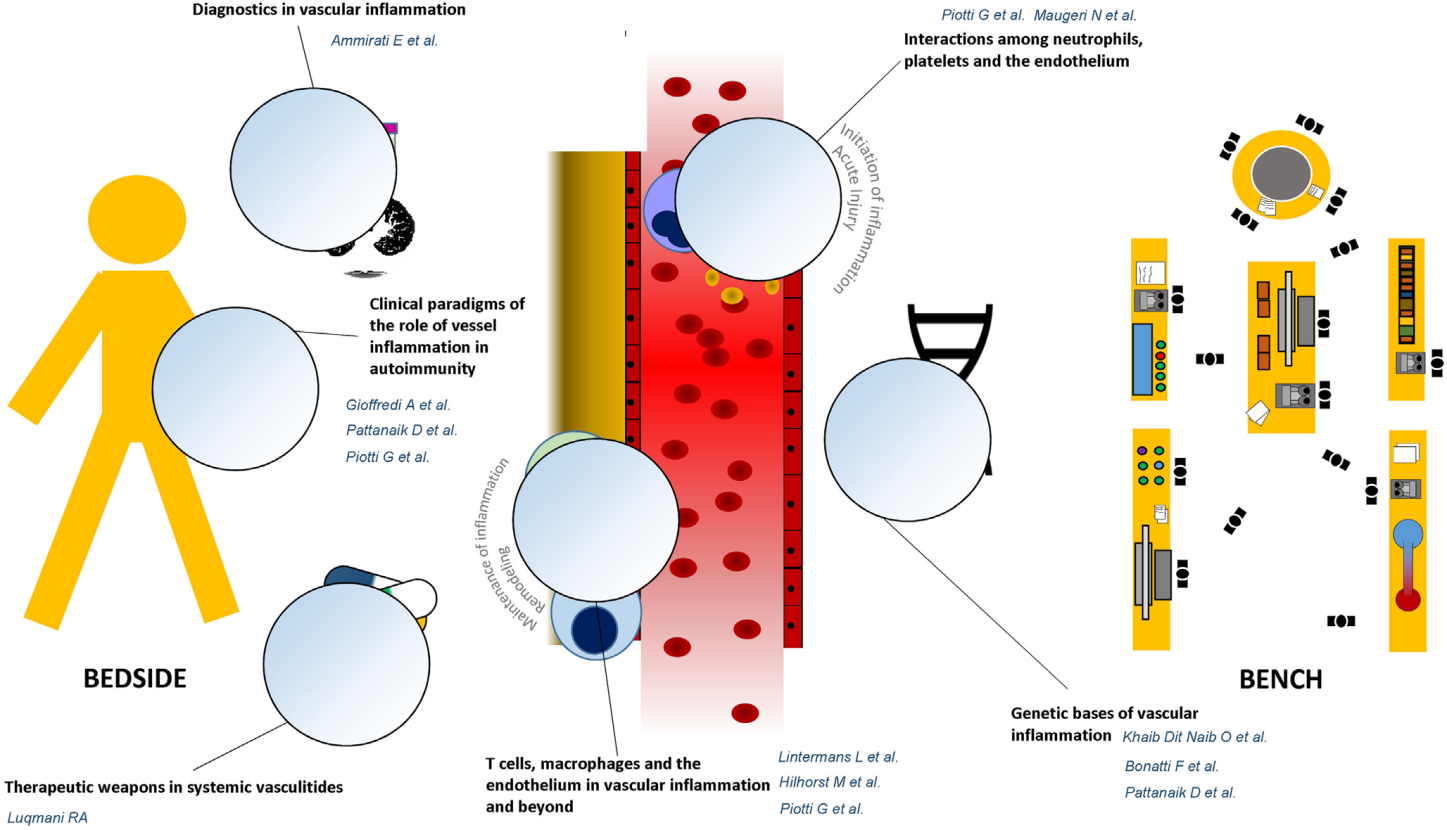
**Graphical guide to the research topic**. The research topic “Vascular inflammation in systemic autoimmunity” is built on a bidirectional cross talk between clinical evidence and unsolved issues emerging from the daily rheumatology practice, at patient’s bedside, and novel discoveries coming from the bench. The Reader is, thus, invited to move from dissertations about the genetic bases of vascular inflammation toward therapeutic and diagnostic applications of most significant innovations in the field of vascular pathophysiology.

Maugeri et al. and Piotti et al. touch the pathophysiology of vascular inflammation and elegantly discuss the role of a tripartite innate immune network composed of the endothelium, platelets, and neutrophils in triggering and maintaining vessel injury as well as thromboembolic complications. In parallel, the articles by Hilhorst et al. and Lintermans et al. taken together constitute a comprehensive, cross-sectional analysis of the role of T-cells and macrophage/T-cells interactions in a wide range of inflammatory conditions, from autoimmune diseases to atherosclerosis.

The article by Piotti and colleagues and the ones by Gioffredi et al. and Pattanaik et al. demonstrate how these core mechanisms deploy in clinical settings, such as renal transplant rejection, eosinophilic granulomatosis with polyangiitis, and systemic sclerosis (Pattanaik et al.; Piotti et al.; Gioffredi et al.). Along with this line, Ammirati et al. discuss how increasing insight about the pathogenesis of vessel inflammation could be translated into clinical practice through the development of novel imaging techniques as well as novel biomarkers. The article by Luqmani moves further from bench toward bedside by reporting most updated therapeutic strategies to contrast vessel inflammation and by critically discussing current therapeutic aims and clinical tools to be considered in the treatment of systemic vasculitides, based on most recent pathogenic achievements.

In summary, we are glad to introduce the reader to this translational research topic on vascular inflammation in systemic autoimmunity, a bird’s eye view on current research trends and, in our mind, a stimulus for discussion and for future deeper investigation on the way the blood vessel and immune cells cooperate to determine vessel injury and inflammation. We really feel that this field – which had been so rewarding for scientists in the last decades – holds promises for major breakthroughs in the next future.

## Author Contributions

All authors contributed in writing and revising the manuscript.

## Conflict of Interest Statement

The authors declare that the research was conducted in the absence of any commercial or financial relationships that could be construed as a potential conflict of interest.
